# Micronutrients in Pediatric Asthma: Biological Mechanisms and Clinical Evidence—A Narrative Review

**DOI:** 10.3390/nu18152567

**Published:** 2026-08-06

**Authors:** Paraschiva Chereches-Panta, Claudia Felicia Pop, Ioana Corina Bocșan, Orlanda Moldovan, Alina-Petronela Bouari-Coblișan, Marcela Daniela Ionescu, Valentina Sas

**Affiliations:** 1 Third Pediatric Discipline, Department 8, Mother and Child, Faculty of Medicine, Iuliu Hatieganu University of Medicine and Pharmacy, 400124 Cluj-Napoca, Romania; pusachereches@umfcluj.ro (P.C.-P.); orlanda.simon@umfcluj.ro (O.M.); sas.valentina@umfcluj.ro (V.S.); 2 Third Pediatric Clinic, Children’s Emergency Clinical Hospital, 400315 Cluj-Napoca, Romania; petronela.coblisan@umf.cluj.ro; 3 General Nursing Discipline, Department 1, Faculty of Nursing and Health Sciences, Iuliu Hatieganu University of Medicine and Pharmacy, 400124 Cluj-Napoca, Romania; felicia.pop@umfcluj.ro; 4 Department of Pharmacology, Toxicology and Clinical Pharmacology, Faculty of Medicine, Iuliu Hatieganu University of Medicine and Pharmacy, 400337 Cluj-Napoca, Romania; 5 Department of Pediatrics, Carol Davila University of Medicine and Pharmacy, 020021 Bucharest, Romania; daniela.ionescu@umfcd.ro; 6 Emergency Clinical Hospital for Children “M.S. Curie”, 041451 Bucharest, Romania

**Keywords:** asthma, vitamin D deficiency, iron deficiency, micronutrients, asthma control, atopy, children

## Abstract

**Introduction:** Asthma is the most common chronic inflammatory disease in childhood, with complex pathogenesis and heterogeneous clinical manifestations. Current efforts are focused on achieving remission through phenotyping and personalized therapy. Micronutrient deficiency is commonly observed in both children and adults, and current evidence points to their role in the pathogenesis of numerous acute and chronic conditions. **Method:** In this narrative review, we analyzed current information regarding the influence of micronutrient deficiencies on asthma control. The aim was to clarify the extent to which current knowledge provides evidence for implementing specific dietary measures in children with asthma to achieve disease control. We conducted a review of the literature covering the past 20 years to assess the impact of iron, vitamin D, and folic acid deficiency on asthma control. The research was performed in PubMed, Web of Science, Cochrane Library and Embase. **Results:** Vitamin D deficiency is associated with the frequency of asthma exacerbations in children. However, data regarding the severity of exacerbations, hospitalizations, or emergency department visits are not consistent across different studies. Vitamin D supplementation has controversial effects on disease control. Iron deficiency may contribute to the development of asthma, but there is no clear evidence. Observational studies have reported associations between iron deficiency, poorer asthma control, frequent exacerbations, and impaired lung function. An association between low folate levels and increased asthma risk and poorer asthma control was also reported. **Conclusions:** Assessing micronutrient status in children with asthma and the effect of correcting deficiencies as part of a personalized treatment plan to achieve symptom control remains a topic that requires further research.

## 1. Introduction

### 1.1. The Burden of Childhood Asthma

Asthma is a complex and heterogeneous condition characterized by chronic inflammation of the airways, which is responsible for bronchial hyperreactivity. Extensive research is constantly being published on the pathogenesis of the disease, diagnostic evaluation, phenotyping, and personalized therapy. Epidemiological data showed, by the end of the last century, an increasing prevalence of allergic diseases, including asthma [1]. Recently, a downward trend in the global burden of the disease over the past 30 years has been reported [2]. The global prevalence of asthma reported in 2020 was 9.1% among children and 11% among adolescents, while adults had a lower prevalence of 6.6% [2]. The mortality rate declined from 1990 to 2019, reaching 13.2% of deaths among children with asthma aged five to nine in the U.S. and 16.4% in the 10–14 age group [3].

Most cases begin in childhood. Approximately half of children with asthma still have poorly or partially controlled symptoms, even in some low- and middle-income countries. This may be due to limited access to essential asthma medications. Any regional or global strategy aimed at improving disease control and even achieving remission is welcome.

Asthma is a relatively common chronic condition in childhood, but concerns regarding its prevention are much more limited compared to other chronic diseases, especially oncological pathology, including leukemia [2]. In the current era, there is a continuous need for changing health policies, promoting healthy lifestyle habits that include a healthy diet and regular physical exercise.

### 1.2. The Role of Atopy in the Pathogenesis of Pediatric Asthma 

There is a complex pathogenic interaction between the development of asthma and atopy due to gene–environment interactions, changes in the microbiome, and epigenetic variations [4]. 

Several prenatal and early-life factors have been associated with an increased risk of developing asthma, although their contribution varies and does not necessarily imply a causal relationship. These include maternal stress, obesity during pregnancy, cesarean delivery, prematurity, low birth weight, and the use of acetaminophen and antibiotics by the mother [5,6]. Tobacco smoke, air pollution, lifestyle, and nutritional factors are also contributing factors. Among the protective factors for healthy development and a low risk of wheezing, high intake of vitamin D or fish oil during pregnancy, early enrolment in preschool, breastfeeding (in some studies), pet ownership, and living on a farm are the most widely studied [6,7,8]. 

Environmental factors are specific risk factors that contribute to the severity and persistence of airway inflammation. Early exposure to allergens is a key pathogenic link associated with a higher risk of developing asthma. Indoor and outdoor airborne allergens are factors that promote airway reactivity and trigger exacerbations by narrowing the airways and promoting their inflammation. Non-allergic airway inflammation also plays an important role in asthma. Asthma exacerbations can be triggered by upper respiratory tract infections, primarily in children. Inhaled irritants (cigarette smoke, vehicle exhaust, certain cosmetics), physical exercise, emotional stress, certain medications, or even foods or beverages may also be associated with asthma attacks.

Genetic susceptibility is now very well characterized. Genome-wide association studies have identified over 400 associations between single-nucleotide polymorphisms and specific traits. Their interaction with various environmental exposures is not specific [6]. The allergic component varies between high-income countries and low- and middle-income countries. A possible link could be the different exposure to environmental allergens, plants, animals, bacteria, or fungi, during the first months of life in a child living on a farm under more precarious socioeconomic conditions. In the Western, industrialized environment, this exposure is reduced, leading to slower and delayed immune maturation, promoting prolonged type 2 immunity and the risk of atopic sensitization and inappropriate immune responses [6,9]. 

Studies report a higher prevalence of allergies among children from high-income social backgrounds [1]. The early onset of the disease in the first years of life may be due both to genetic factors linked to a genetic locus on chromosome 17q21 and to infections with human rhinovirus and respiratory syncytial virus, which are more common in preschoolers [2,9]. Exposure to the microbiota near animal barns and nutritional factors may contribute. One possible mechanism is the reduced ability of dendritic cells during the first 2 years of life to respond to microbial aggression by producing type 1 proinflammatory cytokines and binding to Toll-like receptors (TLRs). In newborns, the type 1 response, T helper (Th)17 and IL-10, is weak, with immune maturation occurring later [10,11]. In addition to environmental factors, the infant’s gut microbiota and lung microbiota also play an important role [6,12]. The risk factors mentioned above can affect the function of the bronchial epithelial barrier and may amplify inflammatory responses. Environmental biodiversity plays a role by limiting IgE sensitization to allergen extracts, while also reducing reactivity to glycoproteins and N-glycans [13,14]. Commensals in the gut microbiota stimulate the production of antimicrobial peptides and induce regulatory T cells and innate cytokines, such as IL-22, which play a role in protecting the intestinal epithelial barrier and maintaining its integrity. [15].

### 1.3. Micronutrients and Common Childhood Disorders

Children’s healthy growth depends on their nutritional status and an adequate intake of macro- and micronutrients. The 2023 Joint Estimates on Child Malnutrition found that 6.8% of children under five suffer from wasting, and 22.3% of the same age group suffer from stunting [16,17]. Micronutrients include vitamins and minerals. They do not provide calories but are crucial for many biological processes [18]. Micronutrient deficiencies are common, particularly in developing countries, and can lead to serious, life-threatening diseases, increasing morbidity and mortality. Micronutrient deficiencies can cause long-term developmental disorders and predispose individuals to other chronic diseases, such as immune-mediated conditions [19,20]. Half of preschool-aged children and two-thirds of non-pregnant women of reproductive age worldwide have micronutrient deficiencies [21].

Insufficient intake of iron, iodine, folate, zinc, or vitamins such as vitamin A, vitamin C, or vitamin B12 can have negative consequences on growth, susceptibility to infections, congenital malformations, cognitive development, and future occupational performance [22,23].

Iron is an important element for the human body, and iron deficiency is the most common nutritional disorder, as one-third of the world’s population suffers from iron deficiency in both industrialized and non-industrialized countries [19,24]. Anemia is a widespread public health concern, particularly in low- and lower-middle-income countries, affecting young children, adolescent girls who are menstruating, and pregnant or postpartum women. It is estimated that 40% of all children aged 6 to 59 months have anemia, with iron deficiency being the most common cause [25].

The main causes of iron deficiency are dietary iron deficiency and inflammation, which lead to reduced iron absorption and utilization [24]. The former results in absolute iron deficiency, in which iron stores are depleted. The latter produces a functional iron deficiency, with an immune response that locks iron into stores and reduces absorption through the involvement of hepcidin in the context of the so-called “nutritional immune response” [19,26,27,28]. The distinction between anemia, classic iron deficiency, and functional iron deficiency—which results from the redistribution of iron to ferritin in the liver and macrophages—is also highlighted in a recent position paper. The latter is essential because its biological effects depend on iron availability rather than on total iron stores [29].

In pediatric patients, iron deficiency can affect not only growth but also lung development [30,31], small intestine function, and cognitive development effects that are irreversible with iron supplementation [19,32]. Iron-deficiency anemia in children is associated with impaired cognitive performance and delayed motor development that may persist in adulthood, along with reduced physical performance and reduced quality of life. Iron deficiency affects DNA replication and the cell cycle, leading to hair loss, nail abnormalities, and oral lesions [33]. The immune response, myelogenesis, and neurotransmission, as well as cytochrome P450 production, can be affected by iron deficiency, leading to increased susceptibility to infections, restless legs syndrome and, possibly, altered drug metabolism [22,34,35]. 

Vitamin D plays a central role in calcium and phosphate metabolism and bone tissue synthesis, and it also has numerous non-skeletal effects. Its availability depends on sun exposure and daily intake, but it can also be influenced by malnutrition, malabsorption syndromes, and genetic disorders. 

In children, the role of vitamin D begins during fetal life and is responsible for normal growth. Low levels of vitamin D during pregnancy are associated with preterm birth, low birth weight, and a predisposition to asthma, type 1 diabetes, autism, or multiple sclerosis. The exact mechanisms underlying vitamin D’s involvement in these processes are not fully understood [18,36,37,38,39,40]. 

Adequate vitamin D levels are essential for bone health and normal immune system function. Low levels of vitamin D are associated with rickets in young children and osteomalacia in older children, causing bone deformities. Vitamin D deficiency may also be linked to the pathogenic mechanisms of autoimmune diseases, metabolic disorders, or cardiovascular processes, and may be connected to cell differentiation and immunoregulation. Research in this field has yielded conflicting results, and evidence of a causal relationship between vitamin D and the prevalence of these disorders has not yet been established [41,42]. 

Other micronutrients associated with normal growth and development are vitamin A, vitamin E, zinc, vitamin C, folic acid, and selenium. Vitamin A deficiency as part of malnutrition has severe consequences on vision causing night blindness, conjunctival lesions, xerophthalmia, or keratomalacia. It is also responsible for cell differentiation and modulation of apoptosis, embryogenesis, and immune function [18,43,44].

Normal development is also regulated by zinc availability, which is involved in cell differentiation, particularly in immune and gastrointestinal tissues [18,45,46]. 

Folic acid is responsible for DNA synthesis, repair, and methylation, playing a central role in cell division and rapid growth [47]. 

## 2. The Link Between the Immune System, Atopy, and Micronutrients 

Micronutrients play important roles in physiological pathways including the immune system response [48], leading to different types of immune-mediated disorders. In recent years, researchers have demonstrated a direct association between allergic disorders and deficiencies in micronutrients such as iron, vitamin D, and zinc. These studies have raised concerns about the actual mechanisms underlying this association, meaning it is not entirely clear whether micronutrient deficiencies influence the immune response or whether immune system disorders that predispose to inflammation are responsible for functional micronutrient deficiency [19,26]. One hypothesis is that micronutrient deficiencies may mimic an infection, activating the innate immune response, which in turn reduces dietary absorption and nutrient availability by sequestering them in storage. This response will also influence the activity of immune cells.

Macrophages play a central role in the immune response through their ability to recognize pathogens, and in homeostasis by facilitating phagocytosis. They also have an important role in iron homeostasis. Macrophages are responsible for iron distribution, being able to recognize nutritional needs and supply tissues with iron [48]. There are two subtypes of macrophages: the pro-inflammatory M1 subtype and the anti-inflammatory M2 subtype. The difference between the two macrophage subtypes also lies in how they manage iron intake. M1 subtypes have less intracellular iron, are not involved in iron distribution, and their activity is less influenced by iron deficiency. In contrast, M2 subtypes have a large intracellular reservoir of metabolically active iron, are responsible for iron distribution, and their activity is strongly influenced by iron deficiency. Iron deficiency can shift M2 activity toward a pro-inflammatory phenotype [19,29,49,50,51]. The possible mechanism involves a reduction in the turnover rate, affecting mitochondrial metabolism and directing macrophages toward hypoxic glycolysis [52]. Iron is also involved in the activity of T-helper cells, with Th1 cells being more sensitive to iron deficiency, a situation in which cytokine activity shifts toward a Th2 pattern [53,54]. Mast cell degranulation is also influenced by iron deficiency; research in this area shows that iron supplementation prevents mast cell degradation [55]. B-cell activity and antibody production are not affected by iron deficiency, but it may promote the activation of cytidine deaminase by modulating the class switch toward immunoglobulin E [52]. Iron is essential for B-cell function and antibody maturation. Iron deficiency may impair immunoglobulin class switch recombination, affecting the production of class-switched antibodies such as IgG and IgA through modulation of AID activity. These alterations in antibody responses may influence immune regulation and allergy susceptibility, although effects on B-cell proliferation and differentiation appear to be context-dependent.

Infections will alter iron homeostasis, leading to a functional iron deficiency and the sequestration of iron in macrophages or the reticuloendothelial system. Although this response reduces viral replication, it will affect immune cell function. The lungs will also be deprived of available iron, supporting the hypothesis that this situation may trigger asthma exacerbations [56]. Vitamin D deficiency may predispose individuals to iron deficiency and is involved in chemotaxis and the activity of monocytes and macrophages, contributing to the stabilization of mast cells and the reduction of mediator release from effector cells [57,58]. 

Furthermore, in addition to the impact of recurrent infections, the exclusion of certain foods from the diets of children with atopic diseases, particularly those with food allergies, may further contribute to nutritional deficiencies. Children with soy and wheat allergies have been reported to be especially susceptible to deficiencies in vitamins, iron, and zinc [59].

Epidemiological studies have shown that children with atopic disorders are eight times more likely to develop iron-deficiency anemia [24,60]. The risk of anemia correlates with the number of comorbid allergic diseases [59]. 

Over the past decade, there has been considerable interest in the primary prevention of asthma, and the role of vitamin D has been extensively studied. Vitamin D deficiency is widespread worldwide [61]. It has been shown to correlate with atopic diseases and elevated levels of eosinophils and immunoglobulin E, though the underlying mechanisms are complex [62,63,64,65,66,67]. 

Single-nucleotide polymorphisms (SNPs) in vitamin D receptors (VDRs) may influence the development of atopy by regulating VDR expression and vitamin D levels [68]. The VDR gene, located on chromosome 12q13.11, is a pleiotropic gene containing nine exons and eight introns. Several SNPs have been described in the VDR gene. Over 900 genes can be transcribed by VDR, which are involved in numerous autoimmune, allergic, and inflammatory diseases. A 2017 pilot study that analyzed various asthma biomarkers and the Taql and Apal genotypes confirmed a higher susceptibility to developing the disease among carriers of these alleles, but no association was found between the genotypes and serum 25OHD levels [69]. 

Some authors have shown that VDR gene polymorphisms, such as *rs7975232*, *rs1544410*, and *rs731236*, can influence signaling pathways involved in modulating the immune system [68] and, consequently, may increase susceptibility to allergic reactions, including asthma [70,71]. Analysis of the impact of VDR SNPs on the regulation of various immune cell activities, including eosinophils, and on the modulation of IgE production by B cells has not demonstrated a direct role for vitamin D. It is associated with total IgE concentrations and markers of eosinophilic inflammation [72,73], but the mechanisms and VDR signaling pathway remain unclear.

A recent case-control study showed that the distribution of VDR SNPs among atopic patients and the control group was uniform, with no statistically significant differences in genotype distribution [74]. The VDR *rs2228570* GG genotype was significantly associated with normal vitamin D levels in both atopic patients and healthy subjects, while the *rs2228570* A allele was associated with low vitamin D levels. The rs3847987 AA and AC genotypes and the A allele were significantly associated with normal vitamin D levels in the control group; however, this association was not observed in patients with asthma and/or atopic dermatitis [74]. In individuals with the VDR *rs11168293* GG genotype, a significant association was found between low vitamin D levels and eosinophilia and elevated total IgE [74]. The T allele of the VDR *rs11168293* gene correlates with normal IgE levels. Similar results were also obtained in previous studies [75,76].

Vitamin D also plays a role in the Th1/Th2 balance and the balance between Th17 cells and regulatory T cells [77,78], with certain VDR polymorphisms potentially influencing vitamin D levels and the risk of developing atopic diseases [79]. S. Rojo-Tolosa et al. demonstrated an association between the Cdx2 polymorphism (rs11568820) and susceptibility to asthma. Other VDR gene polymorphisms included in the analysis, such as *ApaI (rs7975232*), *BsmI (rs1544410)*, *FokI (rs2228570)*, and *TaqI (rs731236)*, were not associated with the risk of asthma [80].

Vitamin D increases the levels of the anti-inflammatory cytokines IL-10, IL-4, and IL-5 and helps reduce pro-inflammatory cytokines such as IL-12, IL-6, IL-8, interferon-γ (IFN-γ), tumor necrosis factor alpha (TNFα), and IL-17 [81,82]. Vitamin D decreases the production of IL-9, IL-5, and IL-8 when memory T helper cells are polarized toward Th9 cells, which play a role in the development of asthma and other allergic diseases [83]. Most research in this field shows that vitamin D deficiency shifts the immune response toward Th2 [81,82,84,85]. However, some studies show that high levels of vitamin D also increase the risk of asthma [86] and correlate with higher levels of total IgE. Pecanha and colleagues demonstrated an association between vitamin D levels and the onset of asthma in early life [87]. Therefore, the relationship with vitamin D levels is not linear [82,88].

Systematic reviews and meta-analyses provide evidence supporting the protective role of higher vitamin D levels in mothers and the risk of developing the disease [89,90,91,92]. In addition to air pollution, maternal and infant malnutrition, and smoking, dietary risk factors play an important role [93]. A diet low in vegetables and fruits, whole grains, milk, omega-3 and omega-6 fatty acids, along with zinc and deficiencies in vitamins A and D, are implicated in morbidity. Among the micronutrients in the mother’s diet implicated in the development of asthma, vitamin D is the most studied; current data demonstrate that genetic polymorphisms in VDR and VDBP genes are associated with an increased susceptibility to asthma [94,95]. Low vitamin D intake in pregnant women is associated with an increased risk of wheezing [96]. Possible mechanisms through which pre- and perinatal vitamin D deficiency exerts its effect include airway contractility, as well as lymphocyte proliferation and cytokine secretion [97,98]. These children exhibit increased airway resistance and a higher prevalence of allergen sensitization [94].

## 3. Methods

We conducted a narrative review of the current literature to evaluate the role of certain micronutrients in the onset and, more importantly, in the control of pediatric asthma. The objective was to identify evidence for the implementation of specific dietary measures in children with asthma to achieve better control of the disease, at a time when there is growing discussion about remission attainment for this chronic condition.

We reviewed the medical literature from the past 20 years regarding the impact of iron, vitamin D, and folic acid deficiencies on asthma control in children. The search was conducted in PubMed/MEDLINE, Web of Science, the Cochrane Library, and Embase. We included original studies, cohort studies, cross-sectional studies, large observational studies, and relevant reviews addressing asthma control and the potential role of micronutrients. We used the following keywords: “vitamin D,” “25-hydroxyvitamin D,” “folic acid,” “anemia,” “iron deficiency,” “ferritin,” “asthma control,” “supplementation,” “child,” “pediatric,” and “adolescent.” We focused on studies involving the pediatric population, excluding those that included only adults, except for those that provided strong arguments in favor of the intended objective and for which no corresponding studies in children were available. Since the studies were highly heterogeneous in terms of both study design and results, the findings were summarized descriptively rather than quantitatively.

## 4. Micronutrients Deficiencies and Their Impact on Asthma in Pediatric Patients

### 4.1. Micronutrients Within the Framework of Precision Medicine in Pediatric Asthma

The findings on VDR polymorphisms support a precision medicine approach to pediatric asthma, suggesting that the clinical effects of vitamin D are influenced not only by circulating 25(OH)D concentrations but also by individual genetic variability. Differences in VDR polymorphisms and genes involved in vitamin D metabolism may explain the heterogeneous associations observed between vitamin D status and asthma susceptibility across ethnic groups and populations. Consequently, vitamin D supplementation is unlikely to provide uniform benefits for all children. Instead, integrating genetic profiling with micronutrient assessment could help identify children who are most likely to benefit from preventive or therapeutic interventions.

Similar principles may apply to other micronutrients. Iron deficiency influences macrophage polarization, Th1/Th2 balance, mast cell activation, and IgE class switching, while zinc participates in epithelial integrity and immune regulation. Because these pathways are modified by both nutritional status and host genetics, the response to micronutrient supplementation is expected to vary between individuals. This variability represents one of the key concepts of precision medicine, in which nutritional interventions are tailored according to biological, genetic, and immunological characteristics rather than applied uniformly.

Overall, current evidence suggests that micronutrients should no longer be viewed solely as environmental modifiers of asthma risk but also as components of an individualized disease phenotype. The interaction between micronutrient status, immune dysregulation, genetic polymorphisms, and environmental exposures contributes to the marked heterogeneity of pediatric asthma. Within the framework of precision medicine, combining nutritional assessment with genetic, immunological, and clinical biomarkers may improve risk stratification, identify children at increased risk of allergic disease, and guide personalized preventive and therapeutic strategies. Although routine genetic testing is not yet recommended in clinical practice, advances in nutrigenomics and pharmacogenomics suggest that individualized micronutrient interventions may become an important component of future pediatric asthma management.

### 4.2. Vitamin D Deficiency 

#### 4.2.1. Correlation with the Onset of Asthma 

Studies over the past decade have sought to provide evidence of the protective role of vitamin D in pediatric asthma. The medical literature is replete with research on prenatal exposure to vitamin D and its relationship to the onset of the disease and on the role of vitamin D as an immunomodulator and anti-inflammatory agent, as well as its contribution to better disease control and optimal lung function. Environmental exposures during pregnancy and early childhood influence the risk of allergic diseases through epigenetic modifications, highlighting the first 1000 days of life as a critical window for both disease susceptibility and preventive interventions [99,100]. The intake of vitamins by a pregnant woman plays an important role and can influence the risk of allergies. For example, B vitamins and vitamin C can affect DNA methylation. Vitamin D has mixed effects on epigenetic programming in the fetus and infant. Vitamin D influences DNA methylation, histone modifications, and gene expression, particularly during the prenatal and perinatal periods, when its deficiency has been associated with an increased risk of atopic dermatitis and asthma [100,101,102]. Vitamin D also enhances anti-inflammatory pathways by modulating glucocorticoid receptor activity and histone acetylation, while reducing airway inflammation through increased HDAC2 expression [100,103]. Collectively, these findings provide a biological basis for the protective role of vitamins A and D against allergic diseases by shaping the epigenetic regulation of immune responses.

A meta-analysis of 15 randomized controlled clinical trials showed that administering high doses of vitamin D to both pregnant women and infants appears to reduce the risk of wheezing and asthma [104]. However, the relationship between vitamin D supplementation in both children and adults remains controversial.

Current data provides new evidence regarding genetic factors. Of the 15,174 SNPs in the VDR gene that have been identified, the most studied polymorphism is *rs2228570*.

Paramonova N. analyzed the effect of these polymorphisms in populations from the Baltic region of Eastern Europe (Latvia and Lithuania) and compared them with data published in studies from the Asian region [105]. The *FokI* and *ApaI* polymorphisms act as risk factors for asthma in the Baltic population, although this has not yet been confirmed for the Asian region. In the subtropical region of Taiwan, both VDR gene variants and 25(OH)vitD concentrations act as risk factors for the development of asthma. In the temperate region of Mongolia, only serum vitamin D levels appear to be a relevant predictor of disease onset. No correlation was found between serum 25(OH)D levels and the *rs2286570 (FokI)*, *rs7975232 (ApaI)*, and *rs731236 (TaqI)* in patients with asthma, although 25(OH)vitD levels are significantly lower in asthmatics compared to the control group [105]. The TC genotype (*rs731236*) was associated with the highest serum levels of 25(OH)vitD and with a protective effect against asthma. A systematic review that included 3495 participants from multiple ethnic groups (1392 with asthma) evaluated the relationship between polymorphisms in the vitamin D receptor (VDR) gene and the risk of developing asthma [103]. It has been shown that the *FokI (rs2228570)* and *TaqI (rs731236)* polymorphisms increase susceptibility to asthma, whereas this association does not hold true for the *ApaI (rs7975232)* and *BsmI (rs1544410)* polymorphisms.

Another systematic review, which included 2491 children with asthma and 3682 control subjects, showed that the *Apa1* and *Taq1* polymorphisms have a protective effect, while *Bsm1* and *Fok1* increase susceptibility to asthma, with ethnic variations observed in the subgroup analysis [106]. An association was observed between the homozygous major allele of *Apa1* and asthma, in contrast to Caucasians and African Americans [76,106]. The results also demonstrated a significant association between polymorphisms in the vitamin D metabolic genes *CYP27A1*, *CYP2R1*, *CYP24A1*, and GC, and the onset of asthma [106]. As long as there is evidence that low vitamin D levels promote allergic hypersensitization and increase the risk of developing asthma, the practical implication is clear and easy to implement.

#### 4.2.2. Correlation with Asthma Control 

Vitamin D deficiency appears to be associated with higher symptom scores and reduced lung function, but research findings are inconsistent.

A 2020 review examined the role of vitamin D deficiency in recurrent wheezing among preschoolers [107]. The results were controversial, particularly when considering the association of respiratory infection as a risk factor for exacerbation [107].

While some authors have shown that in children with lower vitamin D levels there was a negative correlation with the frequency and severity of episodes, as assessed by the need for systemic glucocorticoids [108], other studies found no association with hospitalization, the number of emergency department visits, the need for oral glucocorticoids [87,109], or with oxygen therapy [110] (Table 1). A study by Alyasin et al. showed similar results; vitamin D deficiency, although associated with asthma and low FEV1, was not associated with the risk of asthma exacerbations [111]. A larger population study showed that vitamin D insufficiency was associated with poor asthma control despite inhaled corticosteroid treatment [67]. 

A study examining the relationship between vitamin D and lung function parameters in 71 children aged 7 to 17 years, compared with 77 control subjects, found no association between serum 25(OH)D levels and FEV1 or FVC [112].

**Table 1:** Association of vitamin D status and asthma control.

Author	Year/ Location	Population	Number/ Design	Intervention	Outcome
Alyasin et al. [111]	2011 Iran	Children with asthma	100 case/ control	Vitamin D levels	Low vitamin D levels -associated with the risk of asthma No association with hospitalization for asthma exacerbations
Brehm et al. [39]	2010 USA	Children with mild to moderate persistent asthma	1024	Vitamin D levels vitamin D insufficient ≤ 30 ng/mL	Insufficient vitamin D status was associated with higher odds of any hospitalization or ED visit
Brehm et al. [113]	2012 Puerto Rico	Children Asthma/non-asthma	287/273	Vitamin D levels	Vitamin D insufficiency was associated with -higher odds of asthma exacerbation in the prior year -lower lung function
Ozdogan et al. [112]	2017	Children with asthma	71/cross-sectional case/control	FEV1 FVC Eosinophil count Total IgE Vitamin D	Vitamin D quartiles not associated with lung function
Eroglu et al. [110]	2019 Turkey	Children with recurrent wheezing	52/case/control	Vitamin D levels vitamin D sufficient > 20 ng/mL	No significant relationship between vitamin D levels and hospitalization, oxygen, or steroid therapy

Abbreviations: ED, emergency department; FEV1, forced expiratory volume in first second; FVC, forced vital capacity.

#### 4.2.3. The Effect of Vitamin D Supplementation

In the “Vitamin D Add-on Therapy Enhances Corticosteroid Responsiveness in Asthma” (VIDA) study conducted in adults with asthma, administration of a single dose of 100,000 IU of vitamin D3 was associated with a reduction in the need for inhaled corticosteroids [114]. Given the encouraging findings reported in adults, it is important to examine whether similar benefits have been observed in children with asthma.

In Table 2 we summarized the data on the effect of vitamin D supplementation on asthma control in children. These findings have not yet been translated into clear recommendation for the treatment of asthma or recurrent wheezing in preschoolers, where the trigger is often a respiratory infection and the phenotype is primarily non-eosinophilic asthma [115]. The benefits of vitamin D in modulating the immune response help reduce the frequency of respiratory viral infections and, in this way, contribute to an overall reduction in the frequency of exacerbations and better disease control. In contrast, other studies found that vitamin D supplementation in children with asthma has not been shown to reduce the incidence of exacerbations or improve disease control [116]. 

**Table 2:** The effect of vitamin D supplementation on asthma control in children.

Author	Year/ Location	Population	Number of Patients	Intervention	Outcome
Urashima et al. [117]	2010 Japan	School children with asthma	334	Vitamin D 1200 IU/d 3 months versus placebo	Fewer asthma attacks in children receiving vitamin D compared with children receiving placebo
Majak et al. [118]	2011 Poland	Children with asthma	48	Vitamin D 500 IU/d 6 months versus placebo	-Low serum vitamin D was associated with eight times higher risk of asthma exacerbation -Linear correlation between baseline vitamin D serum level and baseline ATAQ score Asthma exacerbations were significantly reduced in the steroid + Vitamin D group
Madhu Yadav et al. [119]	2013 India	Children with asthma	100	Vitamin D 60,000 IU/month 6 months versus placebo	-Reduced the number of exacerbations -PEFR significantly increased in the treatment group -Reduced the requirement of steroids and emergency visits
Tachimoto et al. [120]	2016 Japan	Children with asthma	89	Vitamin D 800 IU/day 2–6 months versus placebo	-Asthma control test scores were significantly improved in the vitamin D group
Kerley et al. [121]	2016 Ireland	Children with asthma	44	Vitamin D 2000 IU/day 15 weeks versus placebo	-No significant difference between groups regarding subjective asthma control. -A significant decrease in school days missed due to asthma
Ducharme et al. [122]	2019 Canada	Children with viral-induced asthma	47	Vitamin D 2 oral doses of 100,000 IU 3.5 months apart (fall and winter) versus placebo	Patients experienced 2–3 viral-induced asthma exacerbations, with no significant group difference
Forno et al. [123]	2020 USA	Children with asthma	192	Vitamin D 4000 IU 48 weeks versus placebo	Vitamin D_3_ supplementation did not significantly prolong the time to a severe asthma exacerbation, the proportion of participants whose dose of inhaled corticosteroid was reduced, or the cumulative fluticasone dose during the trial
Han et al. [124]	2021 USA	Children with asthma and vitamin D levels below 30 ng/m	176	Vitamin D 4000 IU 48 weeks versus placebo	Vitamin D supplementation was not significantly associated with change in any outcome (lung function measures, asthma control, or asthma-related quality of life)
Jat et al. [116]	2021 India	Children with asthma and vitamin D levels below 20 ng/m	250	Vitamin D 1000 IU/d 9 months versus placebo	No differences in proportion of asthmatic children with ACT score ≥ 20, of asthma exacerbation or side effects after Vitamin D supplementation vs placebo
Lewis et al. [125]	2021 USA	Children with asthma	30	Vitamin D 1000 IU/d 12 months versus placebo	Vitamin D supplementation did not affect the ACT score or FEV1
Thakur [126]	2021 India	Children with moderate persistent asthma	60	Vitamin D 2000 IU/d 12 weeks versus placebo	No significant difference between the two groups in terms of the ACT score, FEV_1_, FeNO, number of exacerbations, emergency visits, hospital admissions, and adverse outcomes.

Abbreviations: ACT, asthma control test; ATAQ, Asthma Therapy Assessment Questionnaire; FeNO, fractional exhaled nitric oxide; FEV1, forced expiratory volume in first second; PEFR, peak expiratory flow rate.

In 2020, Forno et al. published the results of the randomized, double-blind, placebo-controlled Vitamin D Kids Asthma (VDKA) study on the impact of vitamin D supplementation on the time to severe asthma exacerbation [123]. The study included 192 participants with low serum 25-hydroxyvitamin D levels ranging from 14 to 30 ng/mL, who were randomized to either the treatment group, which received 4000 IU of vitamin D3 daily, or the control group, which received a placebo. Disease control was assessed using the Asthma Control Test (ACT). The Asthma Control Test (ACT) is a validated patient-reported questionnaire that evaluates asthma control based on symptom frequency, activity limitations, nocturnal symptoms, and rescue medication use; however, as a subjective assessment tool, it may be influenced by individual perception of disease severity. There was a non-significant difference in the number of days to a severe exacerbation (240 days in the study group versus 253 days in the placebo group, *p* = 0.63) [123]. In the group that received 4000 IU of vitamin D for 16 weeks, 37.5% of participants experienced at least one severe asthma exacerbation during that period, compared with 34.4% of those in the placebo group. The included patients had partially controlled asthma with an initial mean ACT score of over 19. The cumulative dose of inhaled steroids during the study was similar in the two groups [123].

Previously published reviews show conflicting data. While some authors report a reduction in the rate of asthma exacerbations without an improvement in lung function in patients receiving vitamin D, others demonstrate that FEV1 increases [127,128,129] or even decreases in children [130]. A Cochrane meta-analysis published in 2016 showed that while vitamin D3 supplementation in both adults and children with asthma reduces the incidence of severe asthma exacerbations, it does not lead to better disease control [131]. A meta-analysis of 12 studies involving children with asthma (898 who received vitamin D and 973 who received a placebo) concluded that vitamin D supplementation was not associated with better disease control or improved lung function [132]. However, asthma recurrence was lower among those who received vitamin D supplements.

In addition to vitamin D’s role in the onset of asthma, disease control, and lung function, there is recent evidence of its role in limiting the progression toward airway remodeling in patients with severe asthma. The remodeling process involves TGF-β1 and inflammatory cytokines, which stimulate fibroblast invasion of the airway wall and the deposition of the extracellular matrix in the subepithelial layer. An experimental study demonstrated the antifibrogenic effect of 1,25(OH)_2_D_3_ in human bronchial fibroblasts stimulated with TGF-β1 or TNF-α-IL-1β from subjects with severe asthma [133]. 

The authors analyze the potential role of vitamin D3 supplementation in patients with severe asthma, compared to the control group, in inducing apoptosis/necrosis in hematopoietic fibroblasts. The cells were cultured in specific media, and the presence of necrotic, late apoptotic, and early apoptotic cells was tracked on a scatter plot and compared to intact, live cells. The study demonstrates that treatment of bronchial fibroblasts with vitamin D3 leads to an increase in the number of early apoptotic cells [133]. Evidence showing that 1,25(OH)D3 counteracts the effect of TGF-β1 on the mRNA expression of LUM, BGN, and COL5A1 supports its protective role. Treatment of bronchial fibroblasts with 1,25(OH)_2_D_3_ resulted in a significant increase in VDR mRNA expression and CYP24A1 mRNA expression in asthmatics. Differences in the expression of genetic targets were observed only after the addition of 1,25(OH)_2_D_3_, leading to a significant increase in the mRNA expression of BGN, CCL2, CCL5, and CCL11 in patients compared to controls [133]. Another effect of adding 1,25(OH)_2_D_3_ to patients with asthma whose human bronchial fibroblasts were stimulated with TGF-β1 or TNF-α-IL-1β was a reduction in the overall protein secretion of stimulated fibrogenic markers and CC chemokines.

### 4.3. Iron Deficiency 

#### 4.3.1. Correlation with the Onset of Asthma 

The relationship between asthma and iron availability is sustained by the inverse relationship of incidence and prevalence of asthma and iron status [20,134]. Iron deficiency affects both T cells and B cells. It can affect antibody production, reduce the IgG/IgM response, and amplify the IgE response. The biology of allergens itself is regulated by the availability of iron [29]. The prevalence is higher in young boys and tends to be higher in girls after puberty. In addition, menstruation, with a decrease in iron availability, is associated with a worse outcome of asthma exacerbations [135,136,137,138,139]. Serum ferritin levels were associated with asthma and decreased FEV1 in women aged 20 to 49 years [140].

#### 4.3.2. Correlation with Asthma Control 

There are numerous studies on the correlation between asthma control and iron deficiency, but the results remain controversial, as some studies have failed to demonstrate this link. 

Chang et al. found that children with anemia are more likely to have uncontrolled or severe asthma compared with children with controlled asthma [141]. 

Heba et al., in a study based on pediatric patients with asthma, demonstrated that low iron levels are associated with uncontrolled asthma and a more obstructive pattern on function tests among asthmatic patients [142]. 

Pulmonary function tests in children showed lower parameter levels in asthmatic children with iron deficiency anemia compared with those without anemia [143,144].

### 4.4. Folic Acid

Folic acid plays a key role in DNA synthesis, functioning as a coenzyme in the formation of nucleic acids. Processes such as cell generation, maturation, and tissue repair depend on the availability of folic acid, as does fetal development, with significant implications for the neural tube. Because it is involved in DNA methylation, folic acid can influence the regulation of key genes involved in modulating the immune response; these epigenetic modifications underpin the hypothesis that folic acid may be involved in the pathogenesis of allergies. Maternal folate levels correlate with changes in methylation in offspring in genes involved in development and in immune-related pathways, suggesting that early exposure to folate may help “program” how the immune system responds later in life and its susceptibility to atopic diseases [145,146]. Based on this hypothesis, studies have been conducted on the relationship between folic acid status and the risk of, and the severity of, bronchial asthma; however, the specific mechanism by which folic acid, through its immunomodulatory effect and epigenetic mechanisms, influences the progression of bronchial asthma has not yet been elucidated [147,148].

In the pediatric population, low levels of folic acid have been associated with the presence and severity of bronchial asthma and reduced lung function [149,150]. Nicolson et al. obtained similar data, demonstrating an inversely proportional association between folic acid levels and bronchial asthma, without demonstrating a correlation with changes in lung function [151]. Furthermore, Kotb et al., and Ali et al., demonstrated that serum folate levels were lower in children with asthma compared to healthy controls, and that these levels were negatively correlated with asthma severity and exacerbations, but not with pulmonary function parameters [152,153]. Blatter et al. showed that the combination of folate deficiency and vitamin D insufficiency leads to a significant increase in the risk of severe exacerbations; children with both deficiencies had a nearly eightfold higher risk of developing at least one severe exacerbation compared with those with normal levels of both nutrients [154]. In addition, 1 ng/mL increase in serum folate concentration was associated with a significant reduction in the risk of asthma [155]. 

The National Health and Nutrition Examination Survey revealed an inverse association between serum folate levels and total IgE levels, atopy, and wheezing. Participants with the highest folate concentrations had a significantly reduced risk of allergic sensitization and respiratory symptoms [156]. 

An observational study examined the relationship between serum folate metabolites and asthma in a representative sample of the U.S. population. By assessing serum levels and the ratio of 5-methyltetrahydrofolate, the biologically active form of folate, to unmetabolized folic acid, the authors observed that a higher ratio is associated with a lower risk of asthma and better lung function [147].

The data on folic acid supplementation during pregnancy are contradictory. A meta-analysis of studies published between 2015 and 2024, conducted to evaluate the global prevalence of childhood asthma and its major risk factors, found that maternal folate supplementation was associated with a lower risk of asthma in children [157]. However, these findings are not entirely consistent with more recent evidence. A large cohort study of pre-adolescents reported that folic acid supplementation during the third trimester of pregnancy was associated with 34% higher odds of asthma in pre-adolescence (adjusted OR 1.34) [158]. 

The results of the available studies are inconsistent and do not allow for a definitive conclusion regarding a causal relationship between folate status and the risk of asthma. Although some studies suggest a possible protective effect of elevated folate levels, the current evidence remains insufficient to support specific recommendations for the prevention or management of asthma [159]. Furthermore, the results of observational studies may be influenced by environmental, nutritional, or behavioral factors and do not necessarily reflect a direct causal effect of folate on the development of bronchial asthma [160]. 

## 5. Discussion and Conclusions

**Figure 1:**
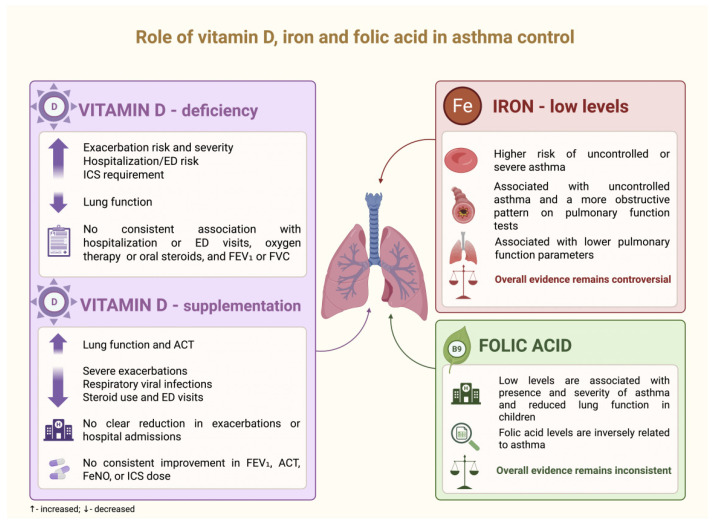
The Role of Vitamin D, Iron, and Folic Acid in Asthma Control. ”Created in BioRender. Moldovan, O. (2026) https://BioRender.com/ena1gfa.” (Accessed on 29 July 2026) [39,87,108,109,110,111,112,113,116,118,119,120,122,123,124,125,126,127,128,131,142,143,144,149,151]. Abbreviations: ED, emergency department; ICS, inhaled corticosteroids; FEV1, forced expiratory volume in first second; FVC, forced vital capacity; ACT, asthma control test; FeNO, fractional exhaled nitric oxide.

The knowledge gained in recent decades regarding the pathogenesis of asthma has enabled better phenotyping of the disease, the identification of underlying molecular endotypes and, consequently, the implementation of personalized treatment plans. The complex role of vitamin D, iron, and folic acid in asthma control is summarized in Figure 1.

The risk of developing asthma is associated not only with serum levels of 25OH vitamin D but especially with polymorphisms in the vitamin D receptor gene. The *ApaI* allele (*rs7975232*) is associated with an increased susceptibility to asthma, while the results for the *TaqI* allele (*rs731236*) remain controversial. The *FokI (rs2228570)*, *BsmI (rs1544410)*, and GC *rs7041* polymorphisms are not associated with the risk of the disease. 

We do not yet have sufficient data to include a recommendation for vitamin D supplementation in the guidelines, nor have we determined the dose needed to improve the course of the disease and achieve remission in pediatric asthma. Dietary recommendations, particularly those regarding iron status and the prevention of micronutrient deficiencies, are not yet part of general asthma management plans.

Iron plays an important role in immune regulation. Iron deficiency may contribute to the development of asthma, but the evidence is not consistent. Several observational studies have reported associations between iron deficiency, poorer asthma control, more frequent exacerbations, and impaired lung function, The link between iron deficiency and asthma could be real, but prospective and interventional studies are needed to confirm it.

Through epigenetic mechanisms, folic acid can influence the expression of genes involved in the immune response and atopy. This mechanism suggests a possible role in the pathogenesis of asthma. Observational studies showed an association between low folate levels and increased asthma risk, poorer asthma control, and more severe disease. Because these results may be influenced by dietary, socioeconomic, and lifestyle factors, it is difficult to confirm a causal effect of folate on the development of asthma.

## 6. Future Perspectives

Highlighting regional and ethnic differences regarding the role of genetic polymorphisms suggests the need for multinational, multicenter studies with a standardized design to elucidate these aspects.

Prospective controlled studies are needed to analyze both the mechanisms and the benefits of vitamin D administration in controlling asthma and improving the quality of life for patients with asthma. Currently, serum vitamin D levels are not routinely measured in children diagnosed with asthma. Such studies could provide additional recommendations regarding the management of children with asthma.

## Data Availability

No new data were created or analyzed in this study. Data sharing is not applicable to this article.

## Abbreviations

The following abbreviations are used in this manuscript:

ACT	Asthma Control Test
ATAQ	Asthma Therapy Assessment Questionnaire
ED	emergency department
FEV1	forced expiratory volume in one second
FeNO	fractional exhaled nitric oxide
FVC	forced vital capacity
GPx	glutathione peroxidase
ICS	inhaled corticosteroids
IFN-γ	interferon-gamma
IL	Interleukin
mRNA	messenger ribonucleic acid
PEFR	peak expiratory flow rate
SNPs	single-nucleotide polymorphisms
TGF-β1	transforming growth factor beta1
TNFα	tumor necrosis factor alpha
TLRs	Toll-like receptors
VDR	vitamin D receptors
